# Crenigacestat (LY3039478) inhibits osteogenic differentiation of human valve interstitial cells from patients with aortic valve calcification *in vitro*

**DOI:** 10.3389/fcvm.2022.969096

**Published:** 2022-09-29

**Authors:** Arseniy A. Lobov, Nadezhda V. Boyarskaya, Olga S. Kachanova, Ekaterina S. Gromova, Anastassia A. Shishkova, Bozhana R. Zainullina, Alexander S. Pishchugin, Alexey A. Filippov, Vladimir E. Uspensky, Anna B. Malashicheva

**Affiliations:** ^1^Almazov National Medical Research Centre, Saint Petersburg, Russia; ^2^Center “Development of Molecular and Cell Technologies”, Research Park, Saint Petersburg State University, Saint Petersburg, Russia

**Keywords:** crenigacestat, LY3039478, calcific aortic valve disease, vascular calcification, valve interstitial cells, osteogenic differentiation, notch inhibitors, gamma-secretase inhibitors

## Abstract

Calcific aortic valve disease (CAVD) is one of the dangerous forms of vascular calcification. CAVD leads to calcification of the aortic valve and disturbance of blood flow. Despite high mortality, there is no targeted therapy against CAVD or vascular calcification. Osteogenic differentiation of valve interstitial cells (VICs) is one of the key factors of CAVD progression and inhibition of this process seems a fruitful target for potential therapy. By our previous study we assumed that inhibitors of Notch pathway might be effective to suppress aortic valve leaflet calcification. We tested CB-103 and crenigacestat (LY3039478), two selective inhibitors of Notch-signaling, for suppression of osteogenic differentiation of VICs isolated from patients with CAVD *in vitro*. Effect of inhibitors were assessed by the measurement of extracellular matrix calcification and osteogenic gene expression. For effective inhibitor (crenigacestat) we also performed MTT and proteomics study for better understanding of its effect on VICs *in vitro*. CB-103 did not affect osteogenic differentiation. Crenigacestat completely inhibited osteogenic differentiation (both matrix mineralization and Runx2 expression) in the dosages that had no obvious cytotoxicity. Using proteomics analysis, we found several osteogenic differentiation-related proteins associated with the effect of crenigacestat on VICs differentiation. Taking into account that crenigacestat is FDA approved for clinical trials for anti-tumor therapy, we argue that this drug could be considered as a potential inhibitor of cardiovascular calcification.

## Introduction

Calcification of the cardiovascular system is a widespread pathology—calcified arteries might be found even in Egyptian mummies (1). Calcific aortic valve disease (CAVD) is one of the most dangerous forms of vascular calcification that caused 102,700 deaths and 12.6 million cases worldwide in 2017 (1, 2). Pathogenesis of CAVD involves the progressive fibro-calcific transformation of aortic valve leaflets, which starts from aortic valve thickening followed by calcification of the valve and this ultimately leads to aortic stenosis. Different stages of CAVD might be found in at least quarter of people older than 65 (3).

Despite such a high frequency in the Eastern world and a high mortality rate, there is no targeted treatment for CAVD yet. As a result, more than 500 thousand aortic valve surgical implantations occur annually all over the world. Only for European medicine, it costs about $13.7 billion and these expenses will only grow with an increasing average age (4). One of the reasons for the absence of target therapy is the difficulty in the development of test systems for analysis of the effects of drugs on CAVD progression *in vitro*. Moreover, there is still no adequate animal model for preclinical study.

Valve interstitial cells (VICs) play a central role in CAVD progression. Normally, these cells maintain valve leaflets homeostasis, but during CAVD progression, they might undergo osteogenic differentiation, which leads to calcific remodeling of valve tissues (5). Thus, inhibition of VICs osteogenic differentiation would be an effective target of anti-CAVD therapy and the effect of probable drugs on VICs osteogenic differentiation might be a fruitful test system for anti-CAVD therapy development.

Dysregulation of Notch-signaling is assumed to be an important factor of CAVD progression (6). Data on the role of the Notch signaling pathway in CAVD is ambiguous. By some data Notch suppress, but by other data stimulate CAVD progression. We have recently shown a dose-dependent action of Notch signaling on osteogenic differentiation—high dosages of Notch suppress, while moderate dosages stimulate osteogenic differentiation (7). We also have previously shown that VICs from CAVD patients are sensitive to proosteogenic stimuli and demonstrate high osteogenic potential when Notch signaling is activated or dysregulated (8, 9). Thus, we assume that inhibition of Notch-signaling in VICs could be considered as a perspective for CAVD treatment. Moreover, some inhibitors of Notch are already at the clinical trial stage in the case of anti-cancer treatment and it would be easy to apply them for anti-CAVD therapy in the case of their efficiency (10). Here we tested two selective inhibitors of Notch signaling pathway (CB-103 and crenigacestat) as inhibitors of osteogenic differentiation of VICs *in vitro*. CB-103 is a small molecule selective inhibitor of the CSL-NICD complex. This complex activates Notch-target genes, so its inhibition leads to interruption of Notch signal transmission (11). Crenigacestat (LY3039478) is a small molecule selective inhibitor of Notch cleavage that suppresses Notch signal transduction by preventing the release of NICD (12). Both drugs have successfully passed the first phase of clinical trials and seem to be promising for treatment (11, 12).

We show here that crenigacestat, but not CB-103, inhibits osteogenic differentiation of VICs without obvious cytotoxicity. Thus, for crenigacestat we additionally found the optimal dosage by experiments with several dilutions and described its probable mechanisms of action by proteomics analysis. The high selectivity of crenigacestat and strong anticalcific effect in non-toxic dosage makes him promising for further preclinical studies.

## Materials and methods

### Cell cultures

Human valve interstitial cells (VICs) were obtained from the tissues of the human aortic valves, which were provided by the Almazov National Medical Research Centre of the Ministry of Health of the Russian Federation. The study was conducted according to the guidelines of the Declaration of Helsinki, and approved by the local Ethics Committee of the Almazov Federal Medical Research Center (ethical permit number 12.26/2014).

Isolation of primary cultures of VICs was carried out by tissue dissociation with collagenase II (2 mg/ml) (13). For further VICs cultivation, we used Dulbecco’s modified Eagle’s medium (DMEM; Gibco, USA) supplemented with 15% of fetal bovine serum (FBS; Gibco, USA), 1% of penicillin/streptomycin (Invitrogene, USA), 1% of L-glutamine (Invitrogene, USA). The culture media was changed twice a week. All cell lines were maintained in a 5% CO_2_ at 37°C. Cells of passage 3–5 were used. The absence of mycoplasma contamination was checked using quantitative polymerase chain reaction (qPCR) according to Janetzko et al. (14). All experiments were carried out in biological triplicates (cells isolated from three donors).

### Osteogenic differentiation

Osteogenic differentiation of VICs was induced by osteogenic medium (DMEM supplemented with 10% of FBS, 1% of penicillin/streptomycin and 50 μM of ascorbic acid, 100 nM of dexamethasone, and 10 mM of β-glycerophosphate (Sigma, USA) (15). For induction of osteogenic differentiation, the cells were plated at 26 × 10^3^/cm^2^ cell density. Other cultivation conditions were the same as for standard cultivation above; osteogenic medium was also changed twice a week.

Calcium deposition was detected by Alizarin Red stain (Sigma, USA) on day 21 of differentiation according to Gregory et al. (16) with minor modifications. The cells were washed with PBS, fixed in 70% ethanol for 60 min, washed twice with distilled water, and stained with Alizarin Red solution. After staining Alizarin Red was extracted by 10% acetic acid and measured by spectrophotometry at 426 nm in Varioskan Lux plate spectrophotometer (Thermo Fisher Scientific, USA). Data processing was performed using Microsoft Excel and GraphPad Prism.

### Inhibitors of osteogenic differentiation

CB-103 and crenigacestat (LY3039478; Medchemexpress, USA) were dissolved in DMSO (dimethyl sulfoxide) to the 100 mM for CB-103 and 10 mM for crenigacestat concentrations.

### Real-time quantitative polymerase chain reaction

For RNA isolation we used an RNA extraction reagent (Eurogen, Russia) in accordance with the manufacturer’s instructions. After isolation, we used 1 μg of total RNA for reverse transcription using the MMLV reverse transcriptase (Eurogen, Russia).

Real-time PCR was performed with SybrGreen qPCR mastermix “qPCRmix-HS SYBR” (Eurogene, Russia) in the LightCycler 96 System (Roshe, Switzerland) according to the following scheme: (1) pre-amplification denaturation of 300 s at 95°C; (2) 45 cycles of three-step amplification (15 s at 95°C, 30 s at 60°C, 30 s at 70°C); (3) the high-resolution melting. Gene expression of RUNX2, GAPDH, HEY1, COL1A1, NOTCH1-3, JAG1 was evaluated 96 h after induction of osteogenic differentiation using specific forward and reverse primers for target genes (Table 1).

**TABLE 1:** Primers used for quantitative polymerase chain reaction (qPCR).

Gene name	Primer	Primer sequence 5′-3′
NOTCH1	F	GAGGCGTGGCAGACTATGC
	R	CTTGTACTCCGTCAGCGTGA
NOTCH2	F	CAACCGCAATGGAGGCTATG
	R	GCGAAGGCACAATCATCAATGTT
NOTCH3	F	TGGCGACCTCACTTACGACT
	R	CACTGGCAGTTATAGGTGTTGAC
JAG1	F	TGCCAAGTGCCAGGAAGT
	R	GCCCCATCTGGTATCACACT
RUNX2	F	GAGTGGACGAGGCAAGAGTT
	R	GGGTTCCCGAGGTCCATCTA
HEY1	F	TGAGCTGAGAAGGCTGGTAC
	R	ATCCCAAACTCCGATAGTCC
GAPDH	F	ACAACTTTGGTATCGTGGAAGG
	R	CAGTAGAGGCAGGGATGATGTT
FURIN	F	CATCATTGCTCTCACCCTGGA
	R	AGTCGTTGGCATTGAGGTGG

### Cell viability

To estimate effect of crenigacestat and CB-103 on cell growth and cell viability we seeded the VICs (for crenigacestat) or HEK-293 cells (for both crenigacestat and CB-103) at 26 × 10^3^/cm^2^ cell density and then treated they with 50, 100, 300, or 500 nM of crenigacestat for 24, 48, or 96 h. After treatment we quantified cell numbers in treated and control wells and performed MTT assay.

Five hundred microliters of MTT solution (0.5 mg/ml) was added to the cultures with further 2-h incubation at 37°C. Then, the cell medium was removed and formazan crystals were dissolved in DMSO. Formazan was measured by CLARIOstar (Labtech, Germany) plate reader at 590 nm.

### Protein isolation

For proteomics analysis, we used VICs at Day 10 after the induction of osteogenic differentiation with osteogenic medium (OM), OM + DMSO, or OM + crenigacestat. The cells were lysed in a Petri dish with RIPA buffer (Thermo Fisher Scientific, USA) supplemented with a complete protease inhibitor cocktail (Roshe, Swithzerland). Cell lysates were stored at –80°C prior to use.

The samples were sonicated and centrifuged (12,000 g, 20 min, 4°C). Proteins were acetone precipitated from the supernatant and washed several times by acetone (EM grade; EMS, USA).

The protein pellet was resuspended in 8 M Urea/50 mM ammonium bicarbonate (Sigma Aldrich, USA). The protein concentration was measured by a Qubit fluorometer (Thermo Fisher Scientific, USA) with “QuDye Protein Quantification Kit” (Lumiprobe, Russia). Twenty micrograms of protein from each sample were used for further analysis.

### “In-solution” digestion

Each sample was analyzed *via* the shotgun proteomics approach. In the first step, the samples were digested by trypsin. Disulfide bonds were reduced and alkylated by incubation of samples with 5 mM DTT (Sigma Aldrich, USA) for 1 h at 37°C with subsequent incubation in 15 mM iodoacetamide (Sigma Aldrich, USA) for 30 min in the dark at room temperature. For tryptic digestion, the samples were diluted with seven volumes of 50 mM ammonium bicarbonate and incubated for 16 h at 37°C with 400 ng of Trypsin Gold (1:50 ratio; Promega, USA). Tryptic peptides were desalted by solid-phase extraction using stage tips. Stage-tips were prepared according to Matamoros et al.: polypropylene Vertex pipette tips (200 μl; SSIbio, USA) were filled with four layers of C18 reversed-phase excised from Empore 3M C18 extraction disks (17). The desalted peptides were evaporated in a Labconco Centrivap Centrifugal Concentrator (Labconco, USA) and stored at –20°C prior to analysis.

### LC-MS/MS

Desalted peptides were dissolved in water/0.1% formic acid for further LC-MS/MS analysis. Approximate 1,000 ng of peptides were used for shotgun proteomics analysis in TimsToF Pro mass spectrometer (Bruker Daltonics, Germany) with nanoElute UHPLC system (Bruker Daltonics, Germany). All samples were analyzed in technical triplicates. UHPLC was performed in two-column separation mode with Acclaim™ PepMap™ 5 mm Trap Cartridge (Thermo Fisher Scientific, USA) and Aurora Series separation column with nanoZero technology (C18, 25 cm × 75 μm ID, 1.6 μm C18; IonOpticks, Australia) in gradient mode with 400 nl/min flow rate with 50°C column temperature. Phase A was water/0.1% formic acid, phase B was acetonitrile/0.1% formic acid. The gradient was from 2 to 35% phase B for 25 min, to 40% of phase B for 5 min, to 95% of phase B for 1 minute with subsequent wash with 95% phase B for 15 min.

The separation column was equilibrated with 4 column volumes and a trap column was equilibrated with 10 column volumes before each sample. CaptiveSpray ion source was used for electrospray ionization with 1,600 V of capillary voltage, 3 L/min N_2_ flow, and 180°C source temperature. The mass spectrometry acquisition was performed in automatic DDA PASEF mode with 0.5 s cycle in positive polarity with the fragmentation of ions with at least two charges in m/z range from 100 to 1,700 and ion mobility range from 0.85 to 1.30 1/K0.

### Statistical analysis

The data obtained by qPCR was processed using Microsoft Excel (calculations) and GraphPad Prism (graphs, statistical analysis). Changes in the expression levels of the target genes were calculated as multiple differences using the comparative method ΔΔCT. mRNA levels were normalized to GAPDH and FURIN as house-keepers. The results are presented as an average of biological and technical repeats. Standard Errors of the Mean (SEM) are indicated. Relative expression levels were compared by ANOVA in GraphPad Prism.

Alizarin red stain quantitative data was analyzed by ANOVA in GraphPad Prism. Standard Errors of the Mean (SEM) are indicated.

MTT data was recalculated to relative cell viability relatively to control and analyzed by non-linear regression in GraphPad Prism. Standard Errors of the Mean (SEM) are indicated.

Annotation of LC-MS/MS data was performed in Peaks Xpro software (license to St. Petersburg State University) using human protein SwissProt database (organism: Human [9606]^1^ ; uploaded 02.03.2021; 20394 sequences) and protein contaminants database CRAP (ftp://ftp.thegpm.org/fasta/cRAP; version of 2019-03-04). The database search parameters were: parent mass error tolerance 10 ppm and fragment mass error tolerance 0.05 ppm, protein and peptide FDR less than 1%, two possible missed cleavage sites, proteins with at least one unique peptide were included for further analysis. Cysteine carbamidomethylation was set as fixed modification. Methionine oxidation, acetylation of protein N-term, asparagine/glutamine deamidation were set as variable modifications. The mass spectrometry proteomics data have been deposited to the ProteomeXchange Consortium *via* the PRIDE (18) partner repository with the dataset identifier PXD029632 and 10.6019/PXD029632.

Statistical analysis of proteomics data was performed in R (version 4.1.1) (19). First of all, we performed qualitative analysis—all proteins presented in all three biological and technical replicates were identified and the biological groups were compared by Venn diagram. Then the proteins with NA in more than 85% of samples were removed and imputation of missed values by k-nearest neighbors was performed with further log transformation and quantile normalization. Then we removed the donor batch effect by the “ComBat” function from the “SVA” package with further analysis of differential expression by “limma” package was performed (20).

The main task was to compare VICs after induction of osteogenic differentiation by OM and by OM + crenigacestat. It is known that DMSO has an impact on cell physiology, but we found that this biological effect was strong at the selected time-point (Figure 4). In general, DMSO is considered to have a small impact on cells on at low concentrations, but its cytotoxicity was significantly demonstrated in concentration higher than 1% (21). While crenigacestat was dissolved in DMSO, the effect of DMSO needs to be considered and we included an additional group—OM + DMSO. As a result, we used a design matrix according to Law et al. (22), which includes both differences between samples with Crenigacestat, DMSO, and classic OM: design matrix = Crenigacestat-(DMSO + OM)/2. This leads to a decrease in the number of identified differentially expressed proteins. Nevertheless, these proteins are strongly correlated with crenigacestat effect.

Finally, we performed ordination of samples by sparse partial least squares discriminant analysis (sPLS-DA) in the package “MixOmics.”, “ggplot2” and “EnhancedVolcano” packages were used for visualization. Functional annotation was performed by the “rWikiPathways” package with Gene Ontology.

Reproducible code for data analysis is available from https://github.com/ArseniyLobov/Proteomics-analysis-of-VICs-treated-by-crenigacestat (accessed on 19.08.2022).

## Results

### Crenigacestat inhibits osteogenic differentiation of valve interstitial cells while CB-103 has no significant effect

**FIGURE 1:**
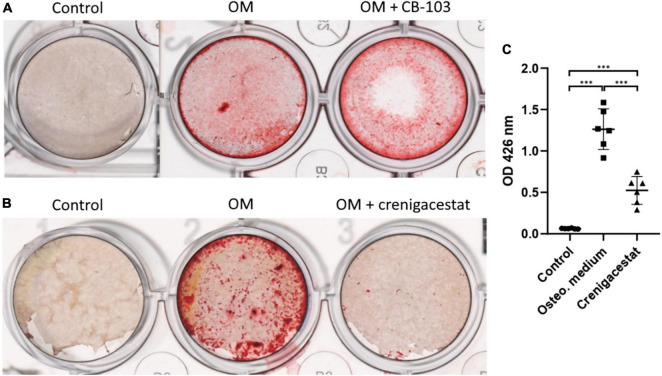
Effect of CB-103 and crenigacestat (LY3039478) on osteogenic differentiation of human valve interstitial cells (VICs) *in vitro*. **(A)** Alizarin Red stain of VICs in control, osteogenic medium (OM), and osteogenic medium supplemented with 100 nM of CB-103 (OM + CB-103). **(B)** Alizarin Red stain of VICs in control, osteogenic medium (OM), and osteogenic medium supplemented with 100 nM of crenigacestat (OM + crenigacestat). **(C)** Quantitative measurement of Alizarin Red stain by spectrophotometry of VICs in control, osteogenic medium (OM), and osteogenic medium supplemented with crenigacestat (OM + cre.). Groups are compared using ANOVA, ^***^P < 0.001.

To reveal the potential anticalcific effect of crenigacestat and CB-103 on VICs we cultured the cells in control, osteogenic medium (OM), and OM supplemented with either crenigacestat or CB-103. On day 21 from the start of differentiation, we stained cells with Alizarin Red to measure the level of calcification of the extracellular matrix (ECM). We found that CB-103 had no significant effects on osteogenic differentiation of VICs (Figure 1A). In contrast, crenigacestat strongly inhibited osteogenic differentiation (Figures 1B,C). The quantitative measurement of Alizarin Red stain by spectrophotometry revealed that the level of calcification was significantly lower in the cells treated with crenigacestat compared to the control cells after osteogenic differentiation alone (Figure 1C).

**FIGURE 2:**
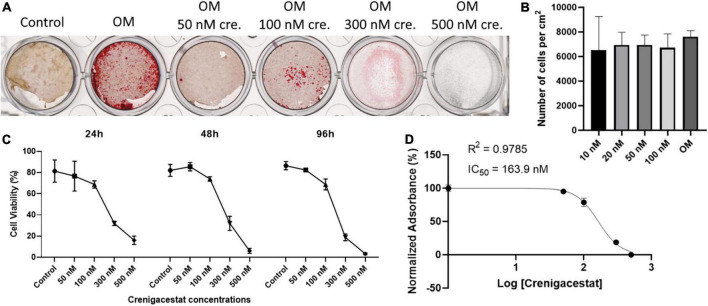
Effect of different dosages of crenigacestat (LY3039478) on survival and osteogenic differentiation of human valve interstitial cells (VICs) *in vitro*. **(A)** Alizarin Red stain of VICs on the 21st day of cultivation in control, osteogenic medium (OM), and osteogenic medium supplemented with different dosages of crenigacestat (cre.) from 50 to 500 nM. **(B)** Boxplot demonstrated the number of VICs cultured in osteogenic media in control conditions or with treatment by different dosages of crenigacestat at 96 h after induction of differentiation and crenigacestat treatment. **(C)** Plot representing effect of different crenigacestat dosages on cell viability relatively to control group in different timepoints. Each point represents an average of three independent replicates with standard error of the mean. **(D)** Sigmoidal curve for MTT assay showing IC50 value of crenigacestat on VICs. Each point represents an average of three independent replicates with standard error of the mean.

To analyze the effect of different dosages of crenigacestat on the VICs we cultured cells under osteogenic conditions with different crenigacestat concentrations from 10 to 500 nM. At the concentrations bellow 20 nM, suppression of osteogenic differentiation was not observed (data not shown). The concentrations from 50 to 100 nM efficiently suppressed the osteogenic differentiation. The concentration from 300 nM caused the death of most cells at the 21st day of cultivation (Figure 2A).

To analyze crenigacestat cytotoxicity, cells were treated with different crenigacestat dosages under cultivation in OM at the 24, 48, and 96 h after treatment and then we quantified cell numbers and performed MTT-test. We found that cell count was not significantly differ between control and concentrations bellow 100 nM (Figure 2B) and we see low cytotoxicity in these concentrations (Figures 2C,D). Concentrations higher than 100 nM have cytotoxic effect (Figure 2C) and IC50 for MTT test on 96 h for crenigacestat is 163.9 nM (Figure 2D).

To compare cytotoxicity of crenigacestat with CB-103 and to compare our data with a previous study on cancer cells we performed MTT-tests for both drugs using HEK-293 cell line (Supplementary File 1). IC50 for MTT test on 96 h for crenigacestat in HEK-293 was 8.8 nM which is 18-fold lower comparing to VICs. CB-103 has lower IC50—2.36 nM at the 96 h.

Thus, crenigacestat is non-toxic in concentrations from 10 to 100 nM. Summarizing these data, the optimal crenigacestat concentration for inhibition of osteogenic differentiation is 100 nM. This dosage was used in further experiments.

### Crenigacestat suppresses Runx2 and Hey1 expression in osteogenic conditions

To confirm that crenigacestat inhibits not only ECM mineralization but also osteogenic differentiation of VICs and to evaluate its effect on Notch signaling we measured the expression Runx2—transcriptional factor regulating osteogenic differentiation.

**FIGURE 3:**
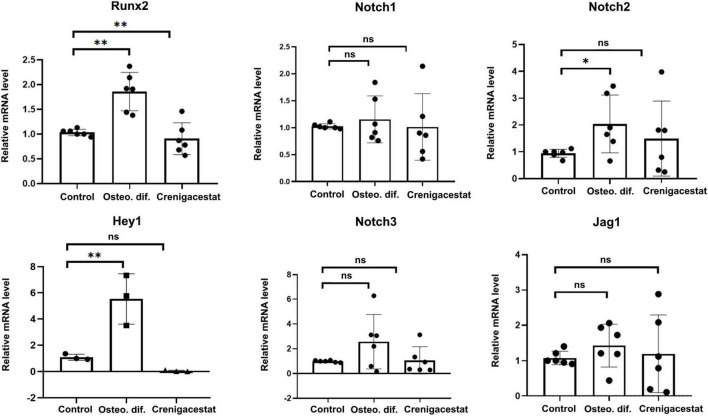
Effect of crenigacestat (LY3039478) on Runx2 (the main regulatory gene of osteogenic differentiation), Hey1 (one of the main Notch target genes) and components of Notch-signaling (Notch1-3, Jag1) in control cells (Control), cells treated with osteogenic medium (OM), and cells treated with osteogenic medium and 100 nM of crenigacestat (OM + cre). Groups were compared using ANOVA, *P < 0.05, ^**^P < 0.01.

The level of RUNX2 expression in cells with inhibited osteogenic differentiation by crenigacestat (OM + 100 nM of crenigacestat) is similar to control cells (Figure 3), while expression of RUNX2 significantly increased in cells cultured in OM were observed normal osteogenic differentiation (Figure 3).

**FIGURE 4:**
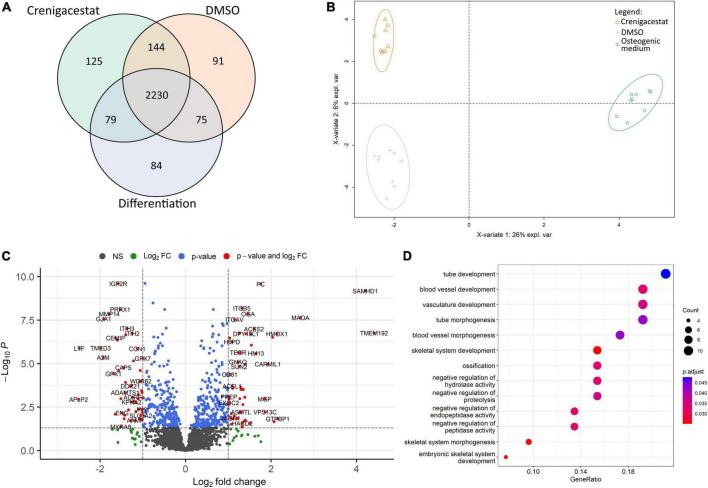
Comparison of proteomics profiles of human valve interstitial cells (VICs) cultured for 10 days in osteogenic medium (OM), OM supplemented with DMSO (DMSO), OM supplemented with 100 nM of crenigacestat (LY3039478) dissolved in DMSO (Crenigacestat). **(A)** Venn diagram representing proteins unique for biological groups compared by shotgun proteomics. **(B)** Partial least-squares discriminant analysis. **(C)** Volcano plot representing differentially expressed proteins (protein names were converted to gene symbols) of VICs cultured with OM supplemented with crenigacestat against VICs cultured with OM and OM supplemented with DMSO. Log2Fold Change, level of change in expression; -Log10P, negative logarithm of the *p*-value. Dotted lines cut off transcripts with *p*-value < 0.05 and Log2Fold Change > |1|. **(D)** Results of Gene Ontology term enrichment analysis of proteins downregulated in VICs by crenigacestat treatment.

Crenigacestat is a selective inhibitor of gamma-secretase and therefore assumed to prevent VICs osteogenic differentiation through suppression of Notch receptor cleavage and Notch signal transduction. To evaluate effect of crenigacestat on Notch we quantified expression levels of Notch components and *HEY1*—Notch target gene known to be activated by Notch in VICs. Crenigacestat suppresses activation of *HEY1* during osteogenic differentiation and has no significant effects of expression of components of Notch signaling (Figure 3).

### Proteomics profile of valve interstitial cells treated with crenigacestat is different comparing to cells in osteogenic medium

To understand molecular mechanisms of inhibition of osteogenic differentiation by crenigacestat we performed proteomics analysis of VICs in OM, OM with crenigacestat dissolved in DMSO, and OM with DMSO 10 days after induction of osteogenic differentiation.

We identified 4,971 proteins. Only 2,828 of them were identified in all technical replicates of all donors at least in one biological group (Figure 4A). For these 2,828 proteins, we performed qualitative analysis and found that each group had unique proteins. The highest number of them were in samples of VICs treated with DMSO (91) and crenigacestat (125; Figure 4A). Similar to those, in the clusterization by sparse partial list discriminant analysis (sPLS-DA) we found differences between VICs in OM and in crenigacestat/DMSO with fewer but still clear differences between crenigacestat and DMSO groups (Figure 4B).

Thus, we performed analysis of differentially expressed genes between VICs in OM supplemented by crenigacestat and VICs in OM. To get a more accurate analysis we used a design matrix which also includes the effect of DMSO. As a result, we revealed 37 differentially expressed proteins, specific for VICs cultured in OM treated with crenigacestat (Figure 4C). Results of analysis of differential expression and proteomics data are represented in the Supplementary File 2.

Functional annotation of differentially expressed proteins by Gene Ontology revealed that proteins upregulated in VICs treated with crenigacestat are associated with fatty acid derivative biosynthetic process (TECR, ACSL1, GGT5, and HACD2) and sulfur compound biosynthetic process (ACSS2, ADI1, TECR, ACSL1, GGT5, and HACD2).

Among downregulated proteins (Figure 4D) one of the most enrichment groups are proteins associated with ossification (MMP14, MYBBP1A, LTF, CCN1, DDX21, COL1A1, GTPBP4, and SPARC), skeletal system development (PRRX1, MMP14, GLA1, LTF, CCN1, COL1A1, MTHFD1L, and ENG), and negative regulation of peptidase activity (ITIH3, ITIH2, C4A, LTF, A2M, GPX1, and APLP2). Interestingly, that 15 out of 52 of downregulated proteins are associated with extracellular exosomes (GO cellular compartment database, *p* = 0.029).

Many of proteins, specific for VICs treated with crenigacestat and found by qualitative comparison were associated with cellular membrane (27 proteins; GO cellular compartment database, *p* = 0.0029). Among top Gene Ontology biological processes were the following: mRNA polyadenylation (four proteins, *p* = 0.00088), protein transport (10 proteins, *p* = 0.0015), autophagy (six proteins, *p* = 0.0021), vesicle-mediated transport (six proteins, *p* = 0.0038), regulation of protein binding (three proteins, *p* = 0.0072), protein processing (four proteins, *p* = 0.013), negative regulation of sphingolipid biosynthetic process (two proteins, *p* = 0.014), smooth endoplasmic reticulum calcium ion homeostasis (two proteins, *p* = 0.014), mesoderm formation (three proteins, *p* = 0.01), endosome organization (three proteins, *p* = 0.02).

## Discussion

Notch signaling is an important factor of osteogenic differentiation of VICs, therefore we tested two inhibitors of Notch signaling for suppression of osteogenic differentiation of VICs isolated from patients with CAVD. As a result, we identified a concentration of crenigacestat, which completely inhibited osteogenic differentiation of VICs *in vitro*, while CB-103 had no significant and reproduced effect on VICs osteogenic differentiation.

### Crenigacestat is a perspective inhibitor of cardiovascular calcification

It was demonstrated, that half-maximal inhibitory concentration (IC50) of crenigacestat is ∼1 nM in most of the tumor cell lines tested (23). Nevertheless, in some other cell lines, crenigacestat was effective in 100 nM–10 μM concentrations for Notch inhibition (24). In our experiments we also found 18-fold differences in IC50 for VICs and HEK-293 (Supplementary File 1). Still, 50–100 nM of crenigacestat was effective for complete inhibition of VICs osteogenic differentiation, which roughly corresponded to the concentrations, which were effective for tumor cells *in vitro* (23, 24). In the MTT test crenigacestat demonstrated no cytotoxicity for VICs in concentrations bellow 100 nM and thus it seems promising for preclinical studies. Nevertheless, it is important to study its effect using other cells—similarly to HEK-293 crenigacestat might be quite cytotoxic for specific cell types. Comparing our data with previous *in vitro* studies, we assume, that dosages of crenigacestat used for anti-tumor therapy might be also effective for CAVD. In clinical trials crenigacestat is generally used in oral form with 25, 50, and 75 mg dosages with optimal oral dosage at 50 mg and maximal dosage at 100 mg (25, 26). It has a controllable toxic (connected manly with gastrointestinal tract) effect without dangerous side effects. In the case of potential therapy against cardiovascular calcification, injections might be more effective compared to peroral form. It has been demonstrated in clinical trials using healthy individuals that intravenous dose of 350 μg of crenigacestat gives only minor side effects (*n* = 39) (26).

Thus, we know the effective dosage of crenigacestat to completely inhibit osteogenic differentiation of VICs *in vitro* and dosage interval which is safe for humans in oral or intravenous injection. We assume that crenigacestat would be effective in clinical trials against vascular calcification, but still argue that additional experiments with tissue (calcified aortic valve) and animal models are necessary.

### Crenigacestat influences the proteins associated with osteogenic differentiation

To understand the molecular mechanisms by which crenigacestat inhibits osteogenic differentiation of VICs we performed shotgun proteomics analysis of VICs treated with crenigacestat at the 10th day after induction of osteogenic differentiation.

Comparing qualitative differences between investigated groups we might emphasize that many of the proteins identified only in VICs treated with crenigacestat are associated with membrane compartment where gamma-secretase (crenigacestat target) is localized. Nevertheless, for most of the identified proteins, we have not found any described interactions with gamma-secretase by the STRING database (data not shown; https://string-db.org/, accessed 08.11.2021) (27). Some of these proteins also might be associated with the inhibition of osteogenic differentiation. For example, sphingolipids might promote osteogenic differentiation, so identified proteins associated with negative regulation of sphingolipid biosynthetic process might be important in inhibition of VICs osteogenic differentiation by crenigacestat (28).

Some of the proteins upregulated by crenigacestat were demonstrated to be upregulated during VICs osteogenic differentiation by previous proteomics and transcriptomics study, e.g., METTL7A, MAOA, SAMHD1, and HTRA1 (29, 30). Moreover, MAOA upregulation in disease-derived population of VICs was found by proteotranscriptomic data and, alongside with METTL7A, assumed to be one of the genes promoting calcification of VICs *in vivo* (31). SAMHD1 was also found to be associated with VICs osteogenic differentiation. Comparison of VICs with various ability to undergo osteogenic differentiation isolated from various donors revealed, that SAMHD1 is one of the markers of calcification-prone VICs (32).

At the same time, crenigacestat causes downregulation of the proteins, specific for osteogenic differentiation, COL1A1 and SPARC as well as downregulation of Runx2 expression and suppression of ECM mineralization. Other downregulated proteins are also associated with osteogenic differentiation, e.g., A2M was identified to be marker of later, calcific stage of CAVD according to spatiotemporal multi-omics data (33); IGF2R is involved in vascular calcification through Erk1/2 and Akt signaling (34); apolipoprotein CIII (APOC3) is associated with CAVD progression (35).

Xu et al. performed single-cell RNA-seq analysis of calcified valve and described cell subpopulations present in the calcific aortic valves. One of the VICs subpopulation segregated by enhanced level of both SPARC and COL1A1 (36), which was downregulated by crenigacestat treatment in our experiments. We, thus, assume that crenigacestat blocks some intermediate stages of VICs osteogenic differentiation.

We demonstrated that anticalcific effect of crenigacestat is associated with suppression of Notch signal transduction. Nevertheless, CB-103 had no anticalcific effect. While crenigacestat is gamma-secretase inhibitor, CB-103 is target to suppression of Notch transcription factor complex (11, 12). Gamma-secretase inhibitors often have off-targets while gamma-secretase, in turn, has a wide list of targets besides Notch (37, 38). Despite the fact, that crenigacestat treatment was associated with suppression of Notch in our experiments, we cannot exclude additional mechanisms, responsible for crenigacestat effect on VICs calcification. Possible mechanisms of crenigacestat action include, but are not limited to, blocking calcium phosphate precipitation, inhibition of alkaline phosphatase or suppression of mineralizing extracellular vesicle release.

The main limitation of our research is the absence of *in vivo* data. The pathophysiology of aortic stenosis is quite unique to humans (39, 40) and the lack of good animal models is currently an important problem of the CAVD field. There are rare examples of animal, in particular mouse, models with quite low penetrance, see recent papers (41, 42). Experimentally induced calcification in VICs *in vitro* is currently considered to be an accurate and affordable model of *in vitro* aortic valve calcification, which is suitable for screening potential pharmacological inhibitors (32). In our experiments we used primary cultures of VICs isolated from calcified aortic valves, i.e., we show the inhibitory effect on diseased human cells. Moreover, we have previously shown that the cells from CAVD patients retain their diseased properties even in *in vitro* cultures (8, 13). Thus, we argue that our data might be extrapolated to the CAVD pathogenesis *in vivo*. Nevertheless, we still consider that *in vivo* experiments in a new model system are necessary to perform in the future. Another important point is that, in opposite to anti-cancer therapy, anti-CAVD therapy suggests lifelong prolonged treatment. Generally, gamma-secretase inhibitors are not suitable for prolonged treatment due to various side effects and such side effects was the main reason for rejection gamma-secretase inhibitors for Alzheimer disease treatment (38). Crenigacestat seems to have much less side effects and assumed to be more suitable for prolonged therapy, but it needs additional studies (12, 24, 26, 38).

## Conclusion

We demonstrated that inhibitor of gamma-secretase crenigacestat (LY3039478) inhibit osteogenic differentiation of human valve interstitial cells isolated from patients with calcific aortic valve disease (CAVD). Working concentrations of crenigacestat did not show significant cytotoxicity. Thus, we suggest that crenigacestat is a perspective drug for a development of therapy against vascular calcification.

## Data availability statement

The datasets presented in this study can be found in online repositories. The names of the repository/repositories and accession number(s) can be found below: the mass spectrometry proteomics data have been deposited to the ProteomeXchange Consortium *via* the PRIDE (18) partner repository with the dataset identifier PXD029632 and 10.6019/PXD029632. Reproducible code for data analysis is available from https://github.com/ArseniyLobov/Proteomics-analysis-of-VICs-treated-by-crenigacestat (accessed on 10.11.2021).

## Ethics statement

The studies involving human participants were reviewed and approved by the Local Ethics Committee of the Almazov Federal Medical Research Center (ethical permit number: 12.26/2014). The patients/participants provided their written informed consent to participate in this study.

## Author contributions

AL and AM: conceptualization. AL, NB, and AM: methodology and validation. AL: software. AL, NB, and OK: formal analysis and visualization. AL, NB, EG, OK, AS, BZ, AP, AF, and VU: investigation. AL and BZ: data curation. AL and NB: writing—original draft preparation. AM: writing—review and editing, and supervision. AM and VU: project administration and funding acquisition. All authors contributed to the article and approved the submitted version.

## Footnotes

1
https://www.uniprot.org/

## Supplementary material

Supplementary File 1 Results of MTT-tests of crenigacestat and CB-103 cytotoxicity using HEK-293 cell line.

Supplementary File 2 Protein expression data (PeaksX Pro output) and results of differential expression analysis.
