# Supplementary material for: Crenigacestat (LY3039478) inhibits osteogenic differentiation of human valve interstitial cells from patients with aortic valve calcification in vitro
Source: Front Cardiovasc Med. 2022 Sep 29;9:969096. doi: 10.3389/fcvm.2022.969096 (PMC9556293; doi:10.3389/fcvm.2022.969096)

Crenigacestat (LY3039478) inhibits osteogenic differentiation of human valve interstitial cells from patients with aortic valve calcification in vitro

Supplementary file 1

Results of MTT-test of cytotoxicity effect of crenigacestat and CB-103 on HEK293 cells

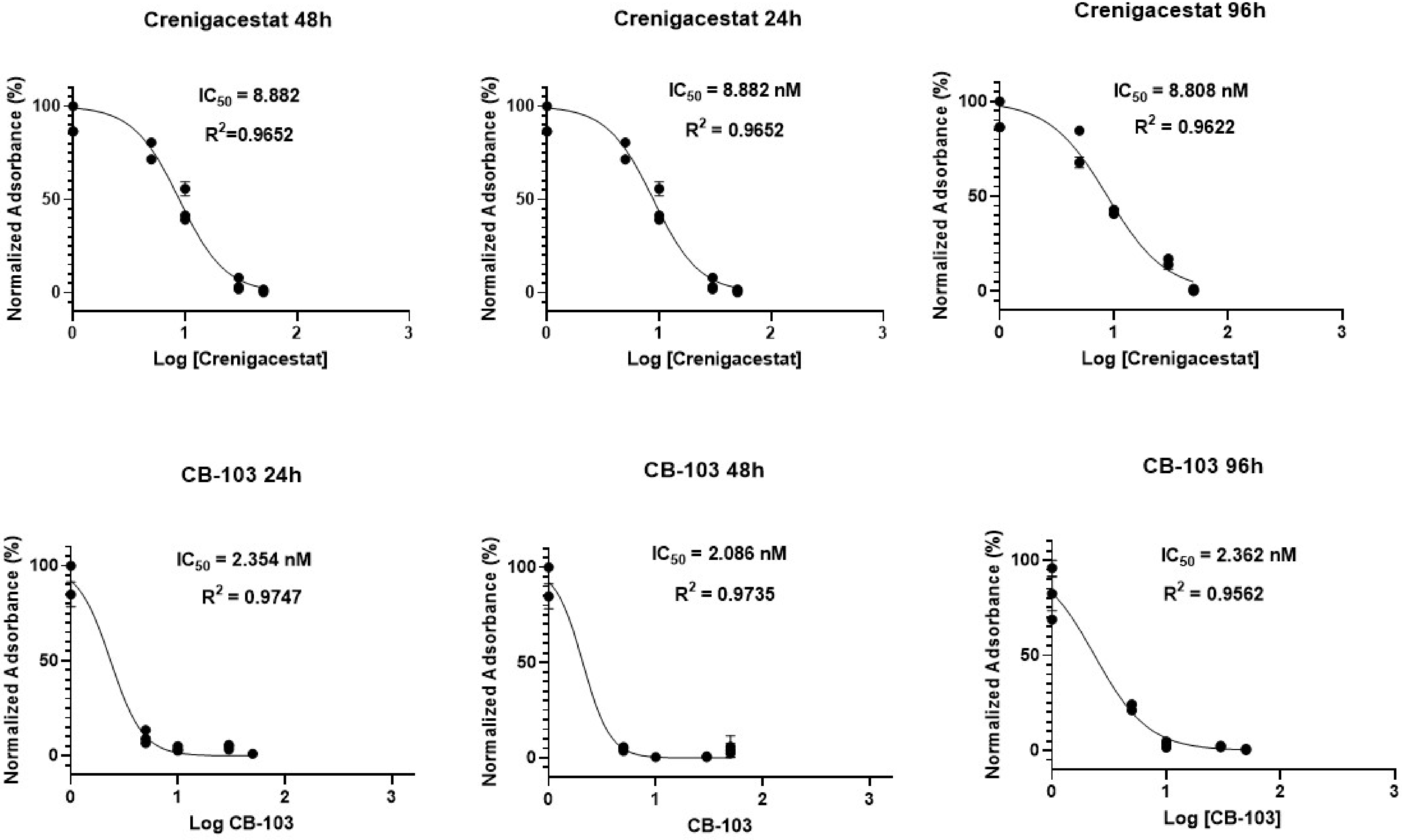

Supplement: Supplementary File 1 — Results of MTT-tests of crenigacestat and CB-103 cytotoxicity using HEK-293 cell line. [file Data_Sheet_1.PDF]
